# The factors predicting pneumonia in COVID-19 patients: preliminary results from a university hospital in Turkey

**DOI:** 10.3906/sag-2005-385

**Published:** 2020-12-17

**Authors:** Selçuk H ÖZGER, Pınar AYSERT YILDIZ, Ümmügülsüm GAYGISIZ, Asiye UĞRAŞ DİKMEN, Zehra DEMİRBAŞ, Mehmet YILDIZ, Esin ŞENOL, Kenan HIZEL, Özlem GÜZEL TUNÇCAN, Kayhan ÇAĞLAR, Gülendam BOZDAYI, Nurdan KÖKTÜRK, Tansu ULUKAVAK ÇİFTÇİ, Gülbin Ş AYGENCEL BIKMAZ, Melda TÜRKOĞLU, Müge AYDOĞDU, Lale KARABIYIK, Abdurrahman TUFAN, Gonca ERBAŞ, Hüseyin Koray KILIÇ, Ayfer KELEŞ, Fikret BİLDİK, İsa KILIÇARSLAN, Mehmet Akif KARAMERCAN, Mehmet Ali ASLANER, Ahmet DEMİRCAN, Mustafa KAVUTÇU, Özlem GÜLBAHAR, Mehmet ARHAN, Hasan BOSTANCI, Hakan TUTAR, Nazlıhan BOYACI DÜNDAR, İpek Kıvılcım OĞUZÜLGEN, Murat DİZBAY

**Affiliations:** 1 Department of Infectious Diseases and Clinical Microbiology, Faculty of Medicine, Gazi University, Ankara Turkey; 2 Department of Anesthesiology and Reanimation, Faculty of Medicine, Gazi University, Ankara Turkey; 3 Department of Public Health, Faculty of Medicine, Gazi University, Ankara Turkey; 4 Department of Clinical Microbiology, Faculty of Medicine, Gazi University, Ankara Turkey; 5 Department of Chest Diseases, Faculty of Medicine, Gazi University, Ankara Turkey; 6 Department of Internal Disease, Faculty of Medicine, Gazi University, Ankara Turkey; 7 Department of Internal Diseases, Rheumatology, Faculty of Medicine, Gazi University, Ankara Turkey; 8 Department of Radiology, Faculty of Medicine, Gazi University, Ankara Turkey; 9 Department of Emergency Medicine, Faculty of Medicine, Gazi University, Ankara Turkey; 10 Department of Medical Biochemistry, Faculty of Medicine, Gazi University, Ankara Turkey; 11 Department of Internal Diseases, Gastroenterology, Faculty of Medicine, Gazi University, Ankara Turkey; 12 Department of General Surgery, Faculty of Medicine, Gazi University, Ankara Turkey; 13 Department of Otolaryngology, Faculty of Medicine, Gazi University, Ankara Turkey

**Keywords:** COVID-19, pneumonia, predicting factors

## Abstract

**Background/aim:**

Pneumonia is the most serious clinical presentation of COVID-19. This study aimed to determine the demographic, clinical, and laboratory findings that can properly predict COVID-19 pneumonia.

**Materials and methods:**

This study was conducted in the Gazi University hospital. All hospitalized patients with confirmed and suspected SARS-CoV-2 infection between 16 March 2020 and 30 April 2020 were analyzed retrospectively. COVID-19 patients were separated into two groups, pneumonia and nonpneumonia, and then compared to determine predicting factors for COVID-19 pneumonia. Variables that had a P-value of less than 0.20 and were not correlated with each other were included in the logistic regression model.

**Results:**

Of the 247 patients included in the study 58% were female, and the median age was 40. COVID-19 was confirmed in 70.9% of these patients. Among the confirmed COVID-19 cases, 21.4% had pneumonia. In the multivariate analysis male sex (P = 0.028), hypertension (P = 0.022), and shortness of breath on hospital admission (P = 0.025) were significant factors predicting COVID-19 pneumonia.

**Conclusion:**

Shortness of breath, male sex, and hypertension were significant for predicting COVID-19 pneumonia on admission. Patients with these factors should be evaluated more carefully for diagnostic procedures, such as thorax CT.

## 1. Introduction

COVID-19 is an epidemic affecting nearly every country, with over 5.5 million confirmed cases and over 350,000 deaths [1].About 80% of patients have mild disease, 20% require hospital admission, and approximately 5% require intensive care admission [2]. In Turkey to date, approximately 160,000 COVID-19 cases were identified and 4500 of these patients died [3].

Pneumonia is the most serious clinical presentation of COVID-19, although the majority of infected patients (80%) experience a mild disease without pneumonia or only mild pneumonia [4]. Several risk factors for severe COVID-19 pneumonia have been well-defined in the literature. However, the factors predicting COVID-19 pneumonia on hospital admission are not yet clear. Advanced radiological methods (CT and USG) are used in addition to chest X-ray [5]. Use of these methods increases labor and cost and causes additional radiation exposure. Therefore, it is important to evaluate all patients for pneumonia-predicting factors on hospital admission and then decide which tests to perform.

In this study we aimed to evaluate the demographic, clinical, laboratory, and imaging findings of our patients to see which factors are predictive of COVID-19 pneumonia on admission.

## 2. Methods

### 2.1. Patients and data extraction

This study was conducted in the Gazi University hospital. All patients with confirmed and suspected SARS-CoV-2 infection hospitalized between 16 March 2020 and 30 April 2020 were analyzed retrospectively. Suspected and confirmed cases were diagnosed according to the criteria of the COVID-19 national guidelines. A suspected case is defined as: (a) fever or at least one of the signs and symptoms of acute respiratory disease (cough and dyspnoea), in which the clinical condition cannot be explained by another cause/disease, and a history of travel abroad 14 days before the onset of symptoms; (b) fever or at least one of the signs and symptoms of acute respiratory disease (cough and dyspnoea) and close contact with a confirmed COVID-19 patient 14 days before the onset of symptoms; (c) fever and at least one of the signs and symptoms of acute respiratory disease (cough and dyspnoea) and hospitalization due to severe acute respiratory infections (SARI), when the clinical condition cannot be explained by another cause/disease; and (d) a sudden onset of fever with cough or dyspnoea without rhinorrhea. A confirmed case is defined as any suspected case in which SARS-CoV-2 has been detected by molecular methods [PCR in nasopharyngeal or lower respiratory tract samples (sputum, endotracheal aspirate)] [6].

Patients under 18 years of age were excluded from the study.

This study was approved by the ethical committee of the Gazi University School of Medicine and was conducted according to the Declaration of Helsinki and Good Clinical Practice (25 April 2020, no.: 269).

### 2.2. Data extraction

A trained team of physicians reviewed and collected the data including patient demographics, comorbidities, home medications, contact history, vital signs, initial laboratory tests, initial electrocardiogram findings, radiological findings, inpatient medications, and outcomes.

Diagnosis of COVID-19 was confirmed by positive real-time reverse transcriptase polymerase chain reaction (RT-PCR) in samples obtained from oropharyngeal and nasopharyngeal sites. The PCR tests were performed in the molecular virology laboratory authorized by the Ministry of Health, General Directorate of Public Health. PCR tests were repeated within 24 h to 48 h in patients with a high suspicion of COVID-19 when the initial PCR test was negative. Patients with two consecutive negative PCR results were considered clinically diagnosed cases in the presence of typical radiological findings for COVID-19 in a thorax CT [7]. COVID-19 positive patients were separated into two groups: pneumonia and nonpneumonia. Pneumonia was diagnosed by clinical signs (dry cough, tachypnea, difficulty in breathing) and imaging findings from chest X-rays. Suspected pneumonia cases were confirmed by CT scan. These two groups were compared in terms of factors predicting COVID-19 pneumonia.

### 2.3. Statistical analysis

SPSS software for Windows (version 17, IBM, Armonk, NY, USA) was used to analyze the data. Categorical variables were described as frequency rates and percentages, and continuous variables were described using mean, median, and interquartile range (IQR) values. The fitness of the continuous variables to the normal distribution was evaluated with the Kolmogorov–Smirnov test. Means for continuous variables were compared using independent group t-tests when the data were normally distributed; otherwise, the Mann–Whitney U-test was used. Proportions for categorical variables were compared using the χ2 test, although the Fisher exact test was used when the data were limited. In the univariate analysis, variables that had a P-value of less than 0.20 and were not correlated with each other were included in the logistic regression model. Sex, diabetes mellitus, hypertension, fever, dry cough, shortness of breath, lymphopenia, and CRP, D-dimer, and ferritin levels were included in the logistic regression model. Values with a type-I error level of below 5% were considered statistically significant.

## 3. Results

A total of 247 patients were included in the study. The median age was 40 (IQR 30–52), and 58% were female. A diagnosis of COVID-19 was confirmed by PCR in 70.9% of these patients. Thirty percent of cases had at least one comorbidity. The most common comorbidities were hypertension (36, 14.6%), cardiovascular disease (22, 8.9%), malignancy (21, 8.5%), and diabetes mellitus (19, 7.7%) (Table 1).

**Table 1:** The baseline characteristics of patients on admission.

Total no = 247	No (%) 1
Demographic information	
Age, median (IQR)	40.0 (30–52)
Sex	
Female	116 (58.0)
Male	84 (42.0)
Comorbidities	
Cardiovascular disease	39 (15.8)
Hypertension	36 (14.6)
Coronary artery disease	17 (6.9)
Congestive heart failure	5 (2.0)
Malignancy	21 (8.5)
Diabetes mellitus	19 (7.7)
Chronic obstructive pulmonary disease (COPD)	13 (5.3)
Chronic renal disease	4 (1.6)
Active smoker	65 (26.3)
At least one comorbid disease	73 (29.6)
At least two comorbid diseases	29 (11.7)
Medications	
Anti-hypertensives	34 (13.8)
Angiotensin-converting enzyme (ACE) inhibitors	16 (6.5)
Angiotensin receptor blockers (ARB)	11 (4.5)
Others (beta-blocker, calcium channel blocker)	10 (4.0)
Statins	7 (3.5)
NSAI drugs	5 (2.0)
Steroids	9 (3.6)
Immunosuppressive agents	18 (7.3)
Distribution of cases	
Confirmed cases	175 (70.9)
Clinically diagnosed cases	72 (29.1)

^1^ Percentages are calculated on the total number of patients.

Among the COVID-19 confirmed cases 21.4% had pneumonia. The predicting factors for pneumonia were evaluated between two groups (Table 2). In the multivariate analysis, male sex [OR: 3.51 (95% CI 1.48–10.73), P = 0.028], hypertension [OR: 6.06 (95% CI 1.29–29.41), P = 0.022], and shortness of breath on hospital admission [OR: 4.92 (95% CI 1.22–19.8), P = 0.025] were significant factors predicting COVID-19 pneumonia (Table 3).

**Table 2:** Comparison of confirmed COVID-19 patients with and without pneumonia.

Total no = 175	N (%) 1	N (%) 1	
	Pneumonian = 37	Non-pneumonian = 138	P-value
Age, median (IQR)	50 (40–68)	34 (28–42)	< 0.001
Sex			0.046
Female	16 (43.2)	85 (61.6)	
Male	21 (56.8)	53 (38.4)	
Comorbidities			
Diabetes mellitus	5 (13.5)	3 (2.2)	0.009
Cardivascular disease	13 (35.1)	7 (5.1)	< 0.001
Hypertension	12 (32.4)	6 (4.3)	< 0.001
Coronary artery disease	4 (10.8)	3 (2.2)	0.033
Congestive heart failure	1 (2.7)	2 (1.4)	0.622
COPD	2 (5.4)	2 (1.4)	0.197
Asthma	4 (10.8)	5 (3.6)	0.107
Malignancy	2 (5.4)	2 (1.4)	0.197
Active Smoker	12 (32.4)	20 (14.5)	0.012
≥ 1 comorbidities	17 (45.9)	13 (9.4)	< 0.001
≥ 2 comorbidities	6 (16.2)	5 (3.6)	0.005
Medications			
Anti-hypertensives	12 (32.4)	6 (4.3)	< 0.001
ACE inhibitors or ARB	8 (21.6)	4 (2.9)	< 0.001
Statins	2 (5.4)	1 (0.7)	0.087
NSAI drugs	1(2.7)	0 (0.0)	0.077
Steroids	1 (2.7)	1(0.7)	0.365
Immunsupressive drugs	2 (5.4)	2 (1.4)	0.481
Clinical characteristics			
Fever, > 38 °C	15 (40.5)	17 (12.3)	< 0.001
Dry cough	18 (48.6)	24 (17.4)	< 0.001
Sore throat	4 (10.8)	11 (8.0)	0.593
Nasal congestion	5 (5.4)	3 (2.2)	0.332
Myalgia or fatigue	13 (35.1)	10 (7.2)	< 0.001
Sputum production	5 (13.5)	2 (1.4)	0.003
Headache	5 (13.5)	6 (4.3)	0.041
Shortness of breath	16 (43.2)	11 (8.0)	< 0.001
Diarrhea	5 (5.4)	3 (2.2)	0.332
Duration of symptoms, median (IQR)2	3 (3–6.5)	3 (2–4)	< 0.085
Tachypnea, > 24 breath/min	7 (18.9)	1 (0.7)	< 0.001
Oxygen saturation < 90%	6 (16.2)	0	< 0.001
Requirement of oxygen support at admission	6 (16.2)	0	< 0.001
Laboratory Findings at hospital admission			
Leukocytopenia < 4000 / mm3	20 (54.1)	58 (42.6)	0.216
Lypmphcytopenia < 800 / mm3	10 (27.0)	4 (2.9)	< 0.001
AnemiaFemale: Hgb < 12 g/dLMale: Hgb < 13 g/dL	11 (29.7)	17 (12.5)	0.012
Thrombocytopenia < 150x103 /mm3	5 (13.5)	10 (7.4)	0.261
ALT > 50 U/L	7 (18.9)	4 (3.0)	0.002
AST > 50 U/L	5 (13.1)	2 (1.5)	0.004
Creatinine > 1.1 mg/dL	4 (10.8)	3 (2.2	0.036
LDH > 248 U/L	22 (59.5)	22 (16.5)	< 0.001
Troponin > 5 ng/L	17 (49.5)	15 (11.3)	< 0.001
Prothrombin time (> 14.7 sec)	9 (24.3)	31 (24.0)	0.971
D-dimer > 1000 ng/mL	10 (27.0)	5 (4.3)	< 0.001
Fibrinogen < 200	1 (2.9)	5 (4.3)	0.730
Ferritin > 800 ng/mL	4 (11.8)	2 (1.7)	0.019
Creatine kinase > 170 U/L	11 (32.4)	19 (14.6)	0.017
C-reactive protein > 40 mg/dL	12 (32.4)	6 (4.5)	< 0.001
Procalcitonin > 0.5 ng/mL	1 (2.7)	5 (3.8)	0.751
Outcomes			
ICU care	7 (18.9)	0 (0.0)	< 0.001
Mortality	2 (5.4)	0 (0.0)	< 0.001
Length of hospital stay, median (IQR)	5.5 (4–8.5)	1(1–3)	< 0.001
ICU care	7 (18.9)	0 (0.0)	< 0.001

COPD: Chronic Obstructive Pulmonary Disease, ACE: Angiotensin-converting enzyme, ARB:Angiotensin receptor blockers, NSAI: Non-steroidal anti-inflammatory, AST Aspartate aminotransferase, ALT:Alanine aminotransferase, ICU: intensive care unit1Percentages are calculated on the total number of patients.2Asymptomatic patients were excluded.

**Table 3:** Multivariate analysis of predicting factors for COVID-19 pneumonia.

	B	S.E.	Wald	Sig.	Exp(B)	95% CI	
						Lower	Upper
Male Sex	1.256	0.570	4.849	0.028	3.511	1.148	10.738
Diabetes mellitus	1.007	1.050	0.920	0.337	2.737	0.350	21.416
Hypertension	1.802	0.788	5.224	0.022	6.060	1.293	28.410
CAD	–1.457	1.428	1.041	0.308	0.233	0.014	3.824
Fever	0.836	0.696	1.442	0.230	2.306	0.590	9.021
Dry cough	0.650	0.633	1.053	0.305	1.915	0.554	6.627
Shortness of breath	1.594	0.711	5.024	0.025	4.926	1.222	19.859
Lymphocytopenia < 800 × 10 9/L	0.489	1.092	0.201	0.654	1.631	0.192	13.870
CRP > 40 mg/dL	0.617	0.893	0.477	0.490	1.852	0.322	10.654
D-dimer > 1000 ng/mL	1.444	0.875	2.723	0.099	4.237	0.763	23.541
Ferritin > 800 ng/mL	0.670	1.471	0.208	0.648	1.955	0.109	34.923

## 4. Discussion

In our study, pneumonia was detected in approximately one-fifth of COVID-19 cases. Male sex, hypertension, and shortness of breath on hospital admission were significant factors for predicting COVID-19 pneumonia.

Male gender and older age were identified as risk factors for COVID-19 disease and a severe clinical course [8–10], although in the current study median age and the percentage of male patients were lower compared to previous studies [8,11,12]. Male sex was found to be a significant factor for predicting COVID-19 pneumonia in this study.

Older age and comorbid diseases are associated with a severe clinical course in COVID-19 cases [10,13]. In a recent metaanalysis, 35.6% of COVID-19 cases had at least one comorbid disease [14]. Comorbidities which are most frequently detected in COVID-19 cases and are proven to be associated with poor prognosis are hypertension, cardiovascular diseases, diabetes, COPD, and active smoking, respectively [10,15,16]. In our study, the relationship between these comorbidities and COVID-19 pneumonia was evaluated, and hypertension was significantly more frequent in patients with pneumonia in the multivariate analysis. We should also take into consideration that two-thirds of cases with pneumonia had no comorbid disease.

In addition to hypertension, the potentially beneficial or harmful effects of ACE inhibitors and ARBs used as an antihypertensive at the onset and the severity of SARS-CoV-2 infection have been discussed [17,18]. These drugs may have a protective effect on disease-related lung injury, although they may increase the risk of SARS-CoV-2 infection. In our study, the use of ACE inhibitors and ARBs in patients with COVID-19 pneumonia was higher than in COVID 19 patients without pneumonia. However, ACE inhibitors or ARBs were not included in the multivariate analysis because of their high correlation with hypertension. Therefore, the role of these antihypertensives in predicting pneumonia was not evaluated in this study.

The most common symptoms in COVID-19 patients were fever, cough, fatigue, and myalgia [19,20]. In the first published data from China, fever was noted during hospital admission in more than 80% of cases, while recent data indicate that fever was detected in only half of the cases [12,13,19–22]. The frequency of fever was much lower in mild COVID-19 cases [23]. In our study, the most common symptoms were cough, fever, shortness of breath, myalgia, and fatigue, respectively. All of these symptoms were more common in patients with pneumonia, but only shortness of breath was predictive of COVID-19 pneumonia. Fever was detected in less than half of the cases of COVID-19 pneumonia on admission, and it was thought that fever is not reliable for predicting COVID-19 pneumonia.

In COVID-19 cases, low levels of oxygen saturation (<88%), lymphocytopenia, D-dimer, ferritin, and AST, LDH, and CRP elevations are shown to be poor prognostic factors at different cut-off values ​​in different cohorts [19,24–26]. In our study these prognostic factors were compared between pneumonia and nonpneumonia groups. In spite of a low level of oxygen saturation, lymphocytopenia, AST/ALT, creatinine, LDH, ferritin, CRP, and D-dimer levels ​​were high in the pneumonia group; no significant relationship was found in multivariate analysis. This might be due to the low number of patients who required intensive care support among COVID-19 pneumonia cases in our study.

This study has several limitations. Our data was collected retrospectively from a single center, and the number of cases was limited. Therefore, patients with COVID-19 pneumonia have not been evaluated according to disease severity. In addition, we could not assess the risk factors for poor outcome, because the number of critically ill patients was low.

In conclusion, shortness of breath, male sex, and hypertension were significant factors for predicting COVID-19 related pneumonia on admission. Patients who have these predicting factors should be evaluated more carefully for further diagnostic procedures, such as thorax CT.
